# Deep Transfer Learning Approach for Automatic Recognition of Drug Toxicity and Inhibition of SARS-CoV-2

**DOI:** 10.3390/v13040610

**Published:** 2021-04-02

**Authors:** Julia Werner, Raphael M. Kronberg, Pawel Stachura, Philipp N. Ostermann, Lisa Müller, Heiner Schaal, Sanil Bhatia, Jakob N. Kather, Arndt Borkhardt, Aleksandra A. Pandyra, Karl S. Lang, Philipp A. Lang

**Affiliations:** 1 Department of Molecular Medicine II, Medical Faculty, Heinrich-Heine-University, 40225 Düsseldorf, Germany; Julia.Werner2@med.uni-duesseldorf.de (J.W.); Raphael.Kronberg@hhu.de (R.M.K.); Pawel.Stachura@med.uni-duesseldorf.de (P.S.); 2 Mathematical Modelling of Biological Systems, Heinrich-Heine-University, 40225 Düsseldorf, Germany; 3 Institute of Virology, Medical Faculty, Heinrich-Heine-University, 40225 Düsseldorf, Germany; Philipp.Ostermann@uni-duesseldorf.de (P.N.O.); Lisa.Mueller@uni-duesseldorf.de (L.M.); Schaal@uni-duesseldorf.de (H.S.); 4 Department of Pediatric Oncology, Hematology and Clinical Immunology, Medical Faculty, Center of Child and Adolescent Health, Heinrich-Heine-University, 40225 Düsseldorf, Germany; Sanil.Bhatia@med.uni-duesseldorf.de (S.B.); Arndt.Borkhardt@med.uni-duesseldorf.de (A.B.); AleksandraAnna.Pandyra@med.uni-duesseldorf.de (A.A.P.); 5 Department of Medicine III, University Hospital RWTH Aachen, 52074 Aachen, Germany; Jkather@ukaachen.de; 6 Institute of Immunology, Medical Faculty, University of Duisburg-Essen, 45147 Essen, Germany; KarlSebastian.Lang@uk-essen.de

**Keywords:** SARS-CoV-2, deep transfer learning, deep learning, drug screening, emetine, chloroquine, remdesivir, hydroxychloroquine

## Abstract

Severe acute respiratory syndrome coronavirus-2 (SARS-CoV-2) causes COVID-19 and is responsible for the ongoing pandemic. Screening of potential antiviral drugs against SARS-CoV-2 depend on in vitro experiments, which are based on the quantification of the virus titer. Here, we used virus-induced cytopathic effects (CPE) in brightfield microscopy of SARS-CoV-2-infected monolayers to quantify the virus titer. Images were classified using deep transfer learning (DTL) that fine-tune the last layers of a pre-trained Resnet18 (ImageNet). To exclude toxic concentrations of potential drugs, the network was expanded to include a toxic score (TOX) that detected cell death (CPETOXnet). With this analytic tool, the inhibitory effects of chloroquine, hydroxychloroquine, remdesivir, and emetine were validated. Taken together we developed a simple method and provided open access implementation to quantify SARS-CoV-2 titers and drug toxicity in experimental settings, which may be adaptable to assays with other viruses. The quantification of virus titers from brightfield images could accelerate the experimental approach for antiviral testing.

## 1. Introduction

Severe acute respiratory syndrome coronavirus-2 (SARS-CoV-2) emerged in 2019 as a pathogen responsible for the coronavirus disease 2019 (COVID-19), which in a proportion of cases causes severe symptoms such as shortage of breath and lung failure [1]. SARS-CoV-2 binds to the entry receptor ACE2, which triggers uptake and cleavage by the proteases Cathepsin B and TMPRSS2 [2]. If viruses cause no or low cytopathic effects (CPE), immunostaining is used to determine virus titers [3]. In contrast, viruses with strong CPE can be visualized by staining of residual cells, resulting in plaque forming units [4]. For coronaviruses, plaque assays have been established, but effects depend on the used cell line and the virus strain [5]. SARS-CoV-2 can be quantified using PCR, which is widely used as a specific and effective diagnostic tool [2]. Furthermore, immunostaining of viral proteins including the nucleocapsid of SARS-CoV-2 have been established to detect infection with SARS-CoV-2 in tissue cultures [6]. All of these protocols involve additional procedures such as fixation and staining to quantify SARS-CoV-2 in tissue culture. These assays have been used to screen for antiviral compounds against SARS-CoV-2 infection. Specifically, hydroxychloroquine and remdesivir were found to reduce SARS-CoV-2 propagation in vitro [6,7]. Both compounds have been tested clinically to treat patients infected with SARS-CoV-2. While hydroxychloroquine was shown to reduce SARS-CoV-2 viral load in a small patient cohort [8], there was no beneficial use in post exposure prophylaxis or as a treatment for mild COVID-19, especially when considering severe side effects [9,10,11]. Remdesivir was able to reduce recovery time compared to a placebo group in hospitalized COVID-19 patients but, when applied as a monotherapy, did not decrease the high mortality rate [12]. Taken together, in vitro assays involving SARS-CoV-2 propagation have been successfully used to identify potential novel antiviral compounds.

Machine learning is rapidly advancing in different areas of life sciences. Deep neural networks have been used for image classification. Specifically, convolutional neural networks are multilayered trained with a back-propagation algorithm to classify shapes [13]. In various tasks in biomedical research, pretrained neural networks have been retrained and successfully used for specific tasks. Pre-trained network models are usually trained on a large number of images in the ImageNet database, allowing them to classify these images into many categories. By retraining these networks on a domain-specific task, previously learned out-of-domain features can improve model convergence and accuracy. Images are provided in an input layer and are connected to the consequent layers, resulting in classification of the provided image through a classification and output layer [13]. Previous studies have shown that cancer tissues can be classified and mutations or expression profiles predicted using retraining of the neural network ‘Inceptionv3’ [14,15]. Furthermore, retraining of the network ‘Resnet18’can predict the microsatellite instability from hematoxylin and eosin (H&E) histology samples of patients with gastrointestinal cancer [16]. Moreover, survival of cancer patients can be predicted from histology samples in combination with or without other parameters using convolutional neural networks [17,18,19]. During the SARS-CoV-2 pandemic, neural networks were used to identify pneumonia caused by SARS-CoV-2 from computed tomography (CT) scans [20,21]. However, deep neural networks have not been used to quantify CPE in experimental assays.

Here, we adapt the pretrained neural network ‘Resnet18’ to classify and score images obtained from SARS-CoV-2 cultures. ‘CPEnet’ was able to attribute a higher score to images from SARS-CoV-2 infected Vero cells, while non-infected cells were given a low score. These scores correlated with other readouts tested. Moreover, further training on a ‘CPETOXnet’ included classification of potential toxicities during drug testing. ‘CPETOXnet’ was able to quantify the inhibition of SARS-CoV-2 replication by chloroquine, hydroxychloroquine, remdesivir, and emetine, while simultaneously identifying the toxic in vitro effects of hydroxychloroquine and emetine.

## 2. Materials and Methods

### 2.1. Viruses

SARS-CoV-2 was used as described previously (Sequence Accession Number: EPI_ISL_425126) [22,23]. SARS-CoV-2 was propagated in Vero cells by infection at a multiplicity of infection (MOI) of 0.001. After 72 h, the supernatant was taken and stored as −80 °C until usage.

### 2.2. SARS-CoV-2 Infection of Cells

Vero cells were cultured as previously described [2]. Cells were cultured in Dulbecco’s modified eagle’s medium (DMEM) with the addition of 10% foetal calf serum (FCS), minimal essential amino acids, and Penicillin/Streptomycin at 37 °C and 5% CO_2_. 3 × 10^4^ cells were seeded per well in a 96 well plate one day before infection. On the next day, the medium was changed to the cell culture medium containing different concentrations of remdesivir, PUH71, AUY922 (Luminespib), NVP-HSP990, EC144, PF-0429113, BIIB021, Tanespimycin (MedChemExpress, Monmouth Junction, NJ, USA), emetine, chloroquine, or hydroxychloroquine (Sigma-Aldrich, St. Louis, MO, USA) dissolved in DMSO. Moreover, 12 serial 3.16-fold dilutions with each second equivalent to a 10-fold dilution were used. The cells were infected 20 min later with different MOIs. An overlay composed of equal proportions 2× DMEM and 2% methylcellulose was added 2 h post infection. EC_50_ and CC_50_ were measured using GraphPad Prism.

### 2.3. Immunofluorescence Staining

Two days after infection, the supernatant was discarded and 4% Formalin was added for 30 min. Hank’s buffer containing Triton-X was applied to the cells for 20 min followed by 10% FCS in PBS for 1 h to block unspecific binding sites. The cells were stained with a SARS-CoV-2 Nucleocapsid antibody (2019 nCoV) (Sino Biology Inc., Eschborn, Germany) for 1 h. Following washing, Fluorescein (FITC) conjugated AffiniPure Goat Anti-Rabbit IgG (H+L) (Jackson Immuno Research, Cambridgeshire, UK) was added for 1 h. The cells were washed again and analyzed with the Nikon Eclipse TS100 fluorescence microscope. Pictures were taken with the software NIS-Elements F4.30.01.

### 2.4. Immunofluorescence Staining Data Analysis

Fluorescent images were analyzed with the ImageJ software. Images were changed to 8-bit and the threshold value was adjusted uniformly for each experiment. The particles were analyzed, whereby the percentage of fluorescence was determined.

### 2.5. Deep Transfer Learning

#### 2.5.1. Architecture

ResNet18 (see Figure S1) was chosen for retraining due to the balance between high accuracy and low prediction time [24,25]. This network has been trained on more than a million images and can classify images into 1000 object categories [26]. For each of the classification tasks, the last two layers (classification and output) were retrained using parameters as previously described [16]. To classify CPE in in SARS-CoV-2 cell cultures into a binary classification (CPE or No CPE) the ‘CPEnet’ was trained and score was calculated by summing up the class for each sub image divided by the total subimages

ScoreCPE = ΣCPE-Tiles/ΣTotal-Tiles(1)

The binary ‘IFnet’ classifies immunofluorescence (IF Signal and No Signal) for each input to quantify immunofluorescence on the whole image is as follows:

ScoreIF = ΣIF-Tiles/ΣTotal-Tiles(2)

In addition, a ‘CPETOXnet’ was trained to recognizing cell death in these cultures, which could identify possible toxic effect of compounds being tested (CPE, TOX and No CPE).

ScoreTox = ΣTox-Tiles/ΣTotal-Tiles(3)

#### 2.5.2. Data and Training

The images used all had a resolution of 2560 × 1920 pixels and were divided into 224 × 224 pixel sub-images, as this was the input shape for the ResNet18. The labels of the sub-images were inherited from the images.

‘CPEnet’ was trained on images obtained from 30 negative controls and 32 SARS-CoV 2 infected tissue cultures (MOI: 0.01) from 5 independent experiments using 21/23 for training, 5 for validation, and 4 for testing. ‘IFnet’ was trained on 40 images each taken from negative controls and SARS-CoV-2 infected cells (MOI: 0.03) after immunostaining for the nucleocapsid of SARS-CoV-2 from 4 independent plates using 28 for training, 6 for validation, and 6 for testing. ‘CPETOXnet’ was trained on 72 images each taken from control cells, staurosporine treated cells (5 µM), and SARS-CoV-2 infected cells (MOI: 0.03) from 3 independent plates using 51 for training, 11 for validation, and 10 for testing. Calculations were performed using Matlab R2020a (Mathworks, Natick, MA, USA) on a desktop computer (i5-6500 CPU @ 3.2GHz (Intel), 8GB RAM or a single GPU, Nvidia Quadro P4000). Furthermore, calculations were performed on a high performance computing cluster of the HHU using Python. The source code is available at: https://github.com/MolecularMedicine2/PyQoVi (Available from: 2 April 2021) (Quantification of Virus in images (e.g., SARS-CoV-2)).

#### 2.5.3. Statistical Analyses

Data are expressed as mean ± SEM. Linear regression was calculated with GraphPad Prism. The networks ‘CPEnet’, ‘IFnet’, and ‘CPETOXnet’ were evaluated on a test dataset and the accuracy and the F-score were determined via calculating the confusion matrix. Statistically significant differences between groups in experiments involving more than one time point were determined using two-way ANOVA.

## 3. Results

### 3.1. Retraining of a Convolutional Neural Network to ‘CPEnet’ Predicts a CPE Score for Images

**Figure 1:**
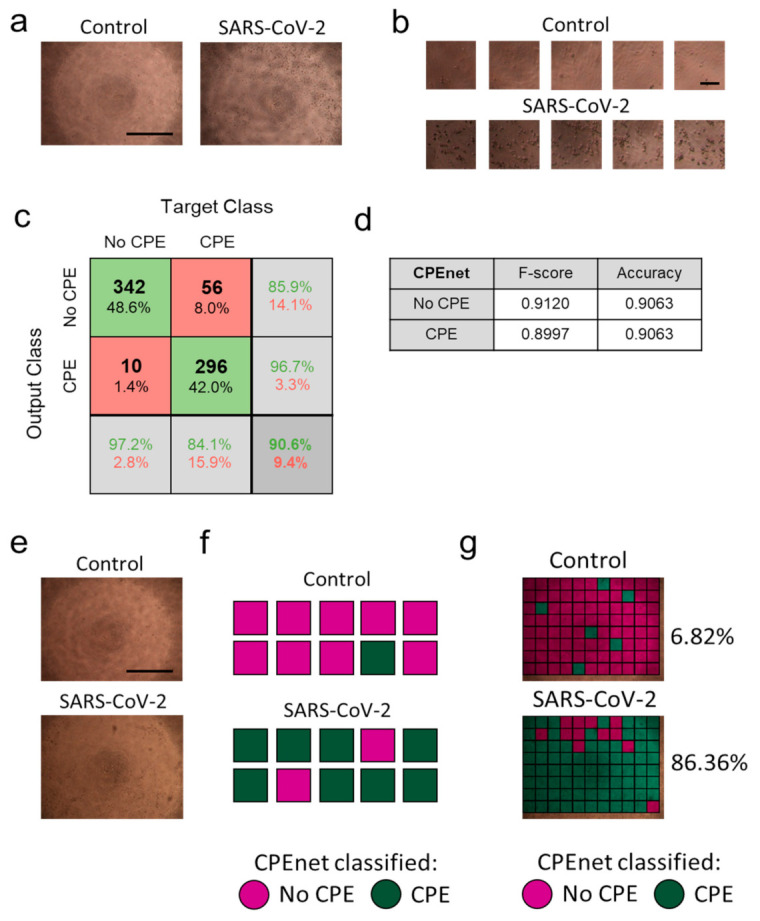
Retraining of ‘Resnet18′ can identify severe acute respiratory syndrome coronavirus-2 (SARS-CoV-2) mediated cytopathic effects (CPE) in live tissue culture brightfield images. Vero cells were infected with SARS-CoV-2 at an multiplicity of infection (MOI) of 0.01. (**a**) Images were taken 3 days after infection (*n* = 32) or without (*n* = 30) infection out of 5 independent experiments (One representative set of images is shown, scale bar = 1 mm). (**b**) Images as in (**a**) were dissected into 224 × 224 image tiles matching the input size of ‘resnet18′ resulting in 1848 training, 440 validation, and 352 testing control image tiles and 2024 training, 440 validation, and 352 testing image tiles from SARS-CoV-2 infected cells (scale bar = 100µm). (**c**) Confusion matrix for the ‘CPEnet’ on the test data set. (**d**) F-score and accuracy on the test dataset. (**e**,**f**) Images from control (upper panel) and SARS-CoV-2 infected (lower panel) tissue cultures were classified by ‘CPEnet’. (**e**) Original images are shown (scale bar = 1 mm). (**f**) Schematic of individual scoring through analysis of image tiles is illustrated. (**g**) Image tiles are stained in red (CPE) or green (no CPE) on the original images as classified by ‘CPEnet’.

The infection of Vero cells with SARS-CoV-2 at an MOI of 0.01 resulted in visible detection of CPE in tissue culture after 72 h (Figure 1a). Images were acquired on live tissue cultures with closed plates. Only one image was taken per well. Accordingly, images showed artefacts originating from condensation and shadows (Figure 1a). To retrain ‘Resnet18′ to detect CPE, we dissected images (2560 × 1920) exhibiting SARS-CoV-2 mediated CPE and negative controls from several experiments into the required input image size (224 × 224) for ‘Resnet18′ (Figure 1b, Figure S1). In total, 30 images of negative controls and 32 images showing SARS-CoV-2 mediated CPE were split into 21/23 images for training, 5 images for validation, and 4 images for testing. Accordingly, 1848/2024 training tiles, 440 validation tiles, and 352 testing tiles were used. Notably, at a MOI of 0.01, CPE was detected in most, but not all image tiles. Hence, we did not expect that all tiles of images from SARS-CoV-2 infected cells would classify as positive. In turn, we expected residual cell death after 72 h of tissue culture in healthy controls resulting in image tiles exhibiting similar features as CPE. Based on these assumptions, image tiles used for training was not used for validation or testing and generated from separate images. In our examples, the averaged F-score to classify CPE on the test dataset was 0.8997, with an achieved accuracy of 0.9063 (Figure 1c,d, Figure S2a). Consistently, when a sample image was classified by ‘CPEnet’ a score of 0.0628 for a negative control and 0.8636 for a positive control was determined (Figure 1e–g). These data indicated that ‘Resnet18′ could be retrained to detect and attribute a number to CPE images in SARS-CoV-2 cultures. Notably, we speculated that the attributed score might reflect the true appearance of image tiles exhibiting CPE regardless of SARS-CoV-2 infection. Hence, further validation is required to investigate whether neural networks can quantify SARS-CoV-2 mediated CPE.

### 3.2. ‘CPEnet’ Generated Quantification of SARS-CoV-2 Cultures Correlates with Immunostainings for the Nucleocapsid of SARS-CoV-2

**Figure 2:**
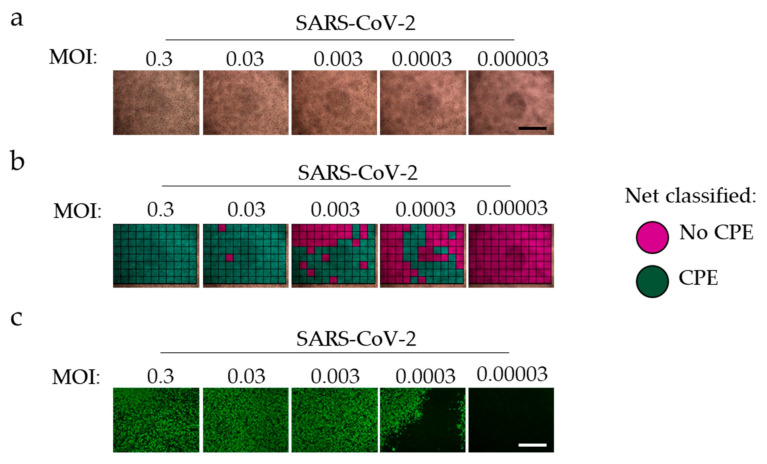

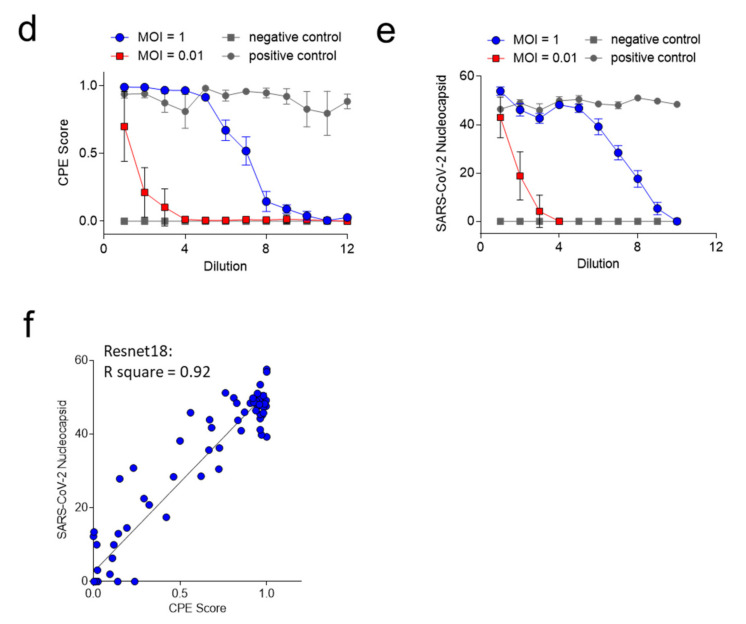
The CPE score correlates with immunofluorescence staining of the SARS-CoV 2 nucleocapsid protein at multiple SARS-CoV-2 titrations. Vero cells were infected with either MOI of 1 or 0.001 followed by serial 3,16-fold dilutions for every second dilution to be 10-fold. (**a**) Brightfield images were taken on day 3 post infection (one MOI = 0.3 representative of *n* = 12 is shown, scale bar = 1 mm). (**b**) Images were classified as indicated by retrained Resnet18. (**c**) 2 days post infection cells were stained with anti-SARS-CoV-2 nucleocapsid antibody (one MOI = 0.3 representative of *n* = 12 is shown, scale bar = 1 mm). (**d**) CPE Score was determined from bright field images on SARS-CoV-2 serial dilutions starting with MOI = 1 (blue line) or MOI = 0.001 (red line). Grey closed circles indicate positive control (MOI = 0.03), closed grey squares indicate negative control (*n* = 4 per well (control); *n* = 12 per well (dilution)). (**e**) Immunofluorescence of nucleocapsid staining of serial dilutions as indicated was quantified using ImageJ (*n* = 4 per well (control); *n* = 12 per well (dilution)). (**f**) Means of the quantification of immunofluorescence from each of 4 repeated experiments as in Figure 2e is shown as a dependence of means of ‘CPEnet’ (*n* = 76).

To determine whether the CPE can reflect data on propagation of SARS-CoV-2, we analyzed plates of titrated SARS-CoV-2 infected cells using other methods of quantification. Notably, CPE is visible 72 h after infection, while we only observed modest CPE in tissue culture 48 h after infection (Figure S3). However, expression of the nucleocapsid SARS-CoV-2 protein can be detected after 48 h. To test the accuracy of ‘CPEnet’, we made 12 serial 3,16-fold dilutions for every second dilution to be 10-fold of SARS-CoV-2 cultures starting with MOIs of 1 and 0.001. As expected, after 72 h, CPE were visible but diminished with increasing dilutions (Figure 2a). The neural network could detect CPE in SARS-CoV-2 infected tissue cultures as well as in immunofluorescence staining of SARS-CoV-2 nucleocapsid protein (Figure 2b,c). While the positive control (MOI 0.03) was classified approximately to a CPE score close to 1, the negative control was attributed a score close to 0 (Figure 2d). Serial dilutions indicated that the input of SARS-CoV-2/1000 appeared 6 dilutions later, indicating that virus titrations could be detected by ‘CPEnet’ (Figure 2d). Notably, we observed a similar pattern with immunostaining for the nucleoprotein of SARS-CoV-2 (Figure 2e). When we correlated the obtained CPE score with the quantification of the immunofluorescence, we found a significant correlation with the R square = 0.92 (Figure 2f).

**Figure 3:**
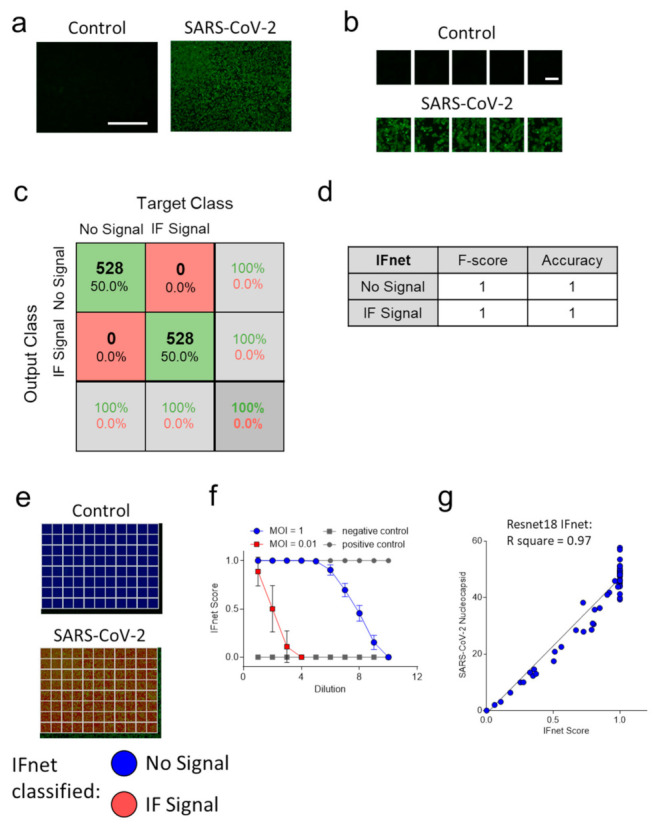
‘IFnet’ can distinguish between infected and noninfected cells with SARS-CoV-2 in images taken after immunofluorescence staining. Vero cells were infected with either MOI of 1 or 0.001 followed by serial 3-fold dilutions. (**a**) Representative fluorescence images are shown as indicated 2 days post infection or without infection, after the cells were stained with anti-SARS-CoV-2 nucleocapsid antibody (*n* = 40, MOI = 0.03, scale bar = 1 mm). (**b**) Images were dissected into 2464 training, 528 validation, and 528 testing 224 × 224 image tiles for each group (scale bar = 100µm). (**c**) Confusion matrix for the ‘IFnet’ on the test dataset. (**d**) F-score and accuracy on the test dataset. (**e**) Fluorescence images from control (upper panel) and SARS-CoV-2 infected (lower panel) tissue cultures were classified by ‘IFnet’. Image tiles are stained in blue (no signal) or red (IF Signal) on the original images. (**f**) IF Score was determined from immunofluorescence images of nucleocapsid staining on SARS-CoV-2 serial dilutions starting with MOI = 1 (blue line) or MOI = 0.001 (red line). Grey closed circles indicate positive control (MOI = 0.03), closed grey squares indicate negative control (*n* = 4 per well (control); *n* = 12 per well(dilution)). (**g**) Means of the quantification of immunofluorescence from each of 4 repeated experiments as in Figure 3e is shown in dependence of means of ‘IFnet’ (Resnet18) predicted IF Score the same experiments (*n* = 76).

Next, we dissected images obtained from the nucleocapsid staining of positive controls (MOI 0.03) and negative controls into training tiles (Figure 3a,b). In total, 28 training images, 6 validation images, and 6 testing images were split into 2464 training tiles, 528 validation tiles, and 528 testing tiles, respectively. Validation and test images were not used for training. We retrained a neural network ‘IFnet’ to detect the proportion of immunofluorescent image tiles with an achieved accuracy of 100% (Figure 3c,d, Figure S2b). In our examples, the averaged F-score to classify IF on the test dataset was 1 (Figure 3e). As expected, the ‘IFnet’ could detect the SARS-CoV-2 titrations (Figure 3f). We found a significant correlation between the ‘IFnet’ score equation (2) and the values obtained from the quantification of the immunofluorescence (Figure 3g) with R square = 0.97. Notably, the images quantified by ‘IFnet’ were the same images used for the quantification of the immunofluorescence, while the quantification of the CPE score was obtained on different plates one day later. Taken together, these data show that neural networks can be used to quantify SARS-CoV-2 cultures with or without immunostaining of viral proteins.

### 3.3. ‘CPETOXnet’ Can Detect Inhibition of SARS-CoV-2 Replication and Identify Toxic Effects In Vitro

**Figure 4:**
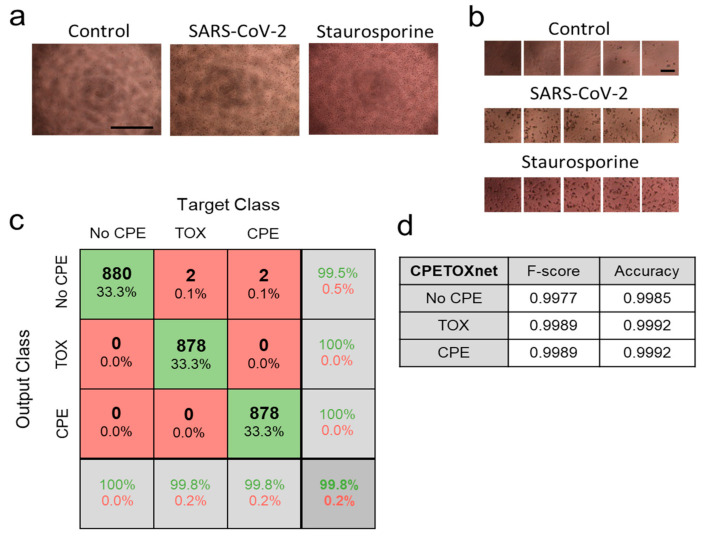

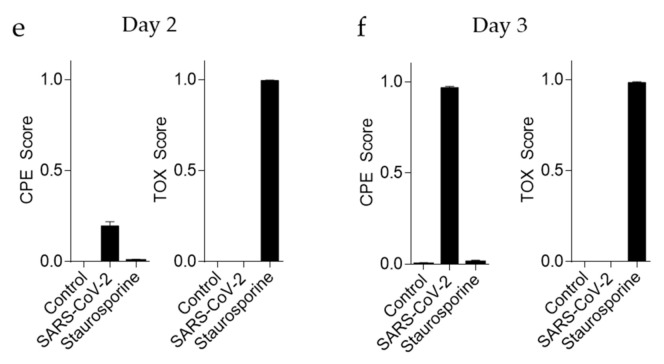
‘CPETOXnet’ can distinguish between CPE and toxicity effects in images from SARS-CoV-2 infected or staurosporine treated cells. Images were taken of uninfected Vero cells, infected Vero cells (MOI of 0.03 with SARS-CoV-2), or treated with staurosporine (5 µM). (**a**) Representative images are shown as indicated 3 days after infection or treatment (*n* = 72, scale bar = 1 mm). (**b**) Images were dissected into 4488 training, 968 validation, and 880 testing 224 × 224 image tiles for each group (scale bar = 100 µm). (**c**) Confusionmatrix for the ‘CPETOXnet’ on the test data set. (**d**) F-score and accuracy on the test dataset. (**e**,**f**) CPE Score (left panels) and TOX Score (right panels) was attributed by ‘CPETOXnet’ to images obtained 2 days (**e**) and 3 days (**f**) after infection (MOI = 0.03) with SARS-CoV-2 or incubation with staurosporine (*n* = 60).

During screening compounds for antiviral effects against SARS-CoV-2, cell toxicity is an important parameter for drug screens. Treatment with staurosporine induces rapid cell death, which can be observed in tissue culture plates (Figure 4a). To confirm the toxicity of staurosporine, we carried out an apoptosis assay on Vero cells with significant differences in comparison to the control group (Figure S4). Accordingly, we dissected 72 images (51 for training, 11 for validation, and 10 for testing) from SARS-CoV-2 infected, staurosporine treated, and control cells into 4488 training, 968 validation, and 880 testing tiles, and retrained a ‘CPETOXnet’, which could predict cell toxicity and CPE (Figure 4b). An overall accuracy of 99.8% on the test data was achieved (Figure 4c, Figure S2c). In our examples, the averaged F-score to classify TOX on the test dataset was 0.9989 (Figure 4d). When we analyzed images taken from tissue cultures at day 2 after infection, ‘CPETOXnet’ could detect CPE in a proportion of image sections. However, since staurosporine already induced severe cell death 2 days after exposure, we observed a high TOX Score (Equation (3)) in these cultures (Figure 4e; Figure S4). Furthermore, when we analyzed images taken at day 3 post infection, ‘CPETOXnet’ reported a high CPE Score only for SARS-CoV-2 infected cells, while toxicity was only attributed to staurosporine treated cells (Figure 4e). These data indicate that ‘CPETOXnet’ can distinguish between late toxicity observed after staurosporine effects and SARS-CoV-2 mediated CPE.

**Figure 5:**
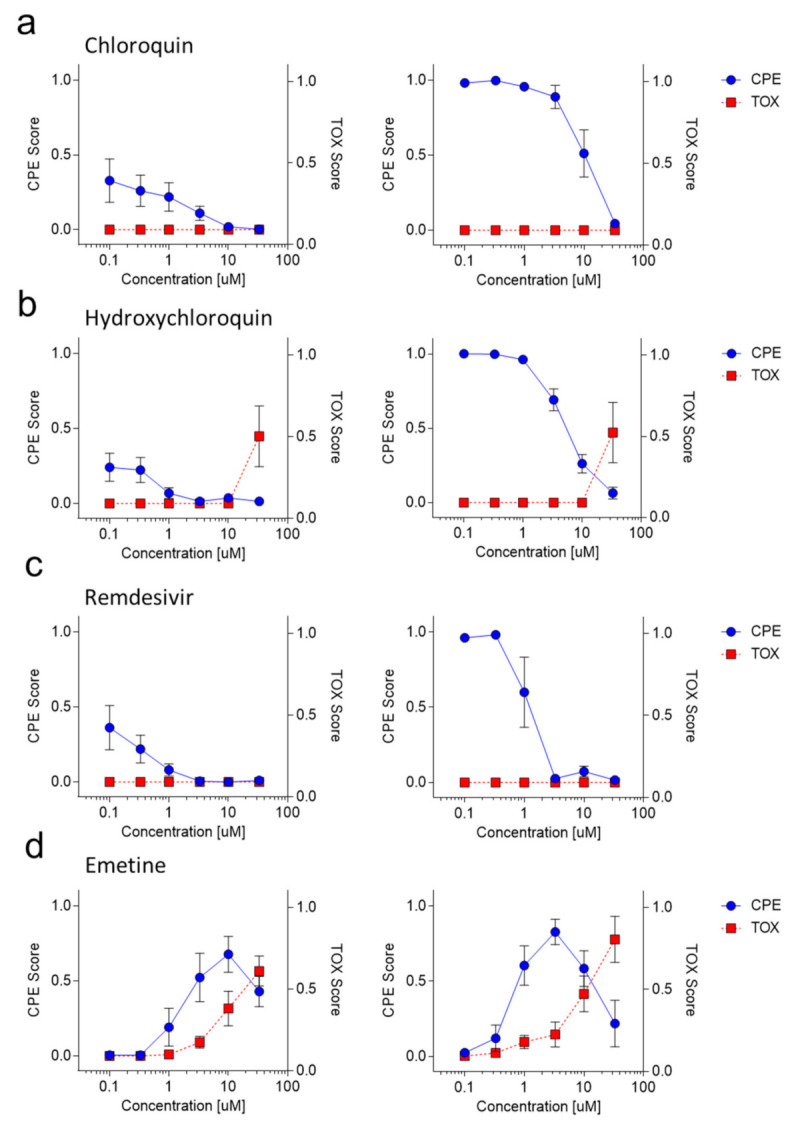
‘CPETOXnet’ can detect toxicity and inhibition of SARS-CoV-2 propagation by compounds in images taken from unstained tissue cultures. (**a**–**d**) Vero cells were treated with the indicated concentration of chloroquine (**a**), hydroxychloroquine (**b**), remdesivir (**c**), and emetine (**d**). Images were taken 2 days (left panels) or 3 days (right panels) after infection with SARS-CoV-2 at an MOI of 0.03. CPE scores (blue) and TOX scores (red) were determined by ‘CPETOXnet’ and are shown in a concentration dependent manner (*n* = 5).

Next, we wondered whether ‘CPETOXnet’ could be used to identify drugs inhibiting SARS-CoV-2 replication. Accordingly, we treated cells with different concentrations of chloroquine, hydroxychloroquine, remdesivir, and emetine, which have been shown to reduce SARS-CoV-2 replication [6,7,27] and DMSO as a control. We monitored cultured cells for 48 and 72 h post infection. As expected, chloroquine was able to reduce the observed CPE at both time points after infection with no observable in vitro toxicity (Figure 5a, EC_50_ = 9.49 µM). Consistently, hydroxychloroquine was also able to reduce SARS-CoV-2 mediated CPE but lead to cell death at the highest concentration (Figure 5b, EC_50_ = 5.27 µM, CC_50_~33.37 µM (out of tested range)). Remdesivir also had antiviral effects against SARS-CoV-2 without toxicity in vitro (EC_50_ = 1.12 µM) while emetine induced toxicity at higher concentrations, which likely also contributed to an increased CPE score in this setting (Figure 5c,d, Figure S5a, CC_50_ = 20.27 µM). Notably, since the observed CPE in these concentrations might reflect cell toxicity, the CC_50_ might be even lower. At lower concentrations emetine was able to limit SARS-CoV-2 replication (Figure 5d, Figure S5a–c, EC_50_ = 0.016 µM). In addition, we screened a library consisting of eight different inhibitors of heat shock protein 90 (HSP90), which has been identified as a protein relevant to SARS-CoV-2 infection [28]. Two days after infection an inhibition with PUH71, AUY922 (Luminespib), NVP-HSP990, EC144, PF-0429113, BIIB021, and Tanespimycin could be visualized, although the CPE could not be detected in all samples at this time point (Figure S6). However, three days after infection the toxicity and CPE score was increased. NVP-HSP990 (EC_50_ = 50.93 µM (out of tested range), CC_50_ = 94.64 µM (out of tested range)), EC144 (EC_50_ = 30.43 µM (out of tested range), CC_50_ = 34.62 µM (out of tested range)), PF-0429113 (EC_50_ = 2.625 µM, CC_50_ = 7.966 µM), BIIB021 (EC_50_ = 9.330 µM, CC_50_ = 10.13 µM (out of tested range)), and Tanespimycin (EC_50_ = 2.086 µM, CC_50_ = 2.940 µM) showed the EC_50_ and CC_50_ to be in close proximity, which suggests a transient effect in this experimental setting and requires further validation and in depth analysis (Figure S6). Taken together, we show that pretrained neural networks can classify SARS-CoV-2 cultures and can assist with quantification during drug screening.

Next, the program was transferred to the open source machine learning framework PyTorch (Python) to enable a wide availability and a more user-friendly handling. As expected, a reanalysis of Figure 2d and subsequent reanalysis of the correlation of the obtained CPE Score analyzed by Python with the quantification of the immunofluorescence (Figure 2e) leads to a significant correlation with R square = 0.91 (Figure S7). The source code is available at: https://github.com/MolecularMedicine2/PyQoVi (Available from: 2 April 2021).

## 4. Discussion

In this study, we showed that retraining a deep convolutional neural network can assist in quantifying SARS-CoV-2 infected cell cultures via bright field images. We retrained a pretrained neural network to classify images from SARS-CoV-2 exposed cells. These images were taken on live, fully covered tissue cultures. Moreover, we retrained a ‘CPETOXnet’ to detect cell toxicity, as well as SARS-CoV-2 mediated CPE. ‘CPETOXnet’ could show the antiviral activity of chloroquine, hydroxychloroquine, remdesivir, and emetine. Furthermore, we demonstrated that hydroxychloroquine and emetine induced dose-dependent cell toxicity in vitro.

Deep neural networks were already used in medical applications to identify and predict mutations in cancer patients [14,16]. Furthermore, neural networks are used to classify CT scans during diagnosis of COVID-19 [20,21]. Our proposed neural network can identify CPE of SARS-CoV-2 cultures on brightfield images taken from closed tissue culture plates. This experimental setting, while very simple, also causes image artefacts through shadows and/or media. These artefacts are observed in almost all image files. Therefore, the individual image tiles can appear different in shape and color. Interestingly, since neural networks are trained on these image tiles, these artefacts are compensated for. ‘CPETOXnet’ could detect toxicity in emetine treated cells but also CPE when emetine was further titrated. This is expected, since lower concentrations would result in modest cell death, which might appear as a CPE. Likewise, this would suggest that strong CPE would be classified as toxicity by ‘CPETOXnet’. Emetine inhibited SARS-CoV-2 mediated CPE at low concentrations suggesting that the classified CPE in emetine treated cells is likely attributed to the toxicity rather than the SARS-CoV-2 induced effects. Accordingly, the CC_50_ might be lower than attributed through the TOX score. These data suggest that detection of cell toxicity needs to be validated with standard techniques. Furthermore, drugs with absence of a TOX score in this experimental setting need to be further tested for cell toxicity. The CPE Score attributed by the convolutional neural network is not specific for SARS-CoV-2. Accordingly, observed effects from a screen have to be verified with specific methods such as quantitative PCR and/or immunofluorescence approaches.

Moreover, this method relies on CPE in brightfield images. Accordingly, when used in our described assay, it will only show antiviral effects of drugs affecting SARS-CoV-2 induced CPE. Specifically, virucidal drugs, virus neutralizing drugs, or drugs affecting viral entry might show a prominent inhibition by using this assay. However, drugs affecting viral replication will only be detected in this experimental setting if CPE in SARS-CoV-2 cultures is inhibited. To assess the effect of drugs on viral replication in depth, infected cells could be washed shortly after infection with collection of the supernatant over time. The supernatant should be used to infect a fresh set of cells to determine the SARS-CoV-2 titer, which could be also performed with the use of ‘CPEnet’.

Although our described approach is simple, it was successfully able to validate compounds that might be useful in early clinical therapy regimens during SARS-CoV-2 infections. Using the described approach, the retrained neural network can be used to detect a variety of effects observed in tissue culture suggesting a broad applicability. Hence, it is tempting to speculate that the described procedures can be used early during an outbreak when there might be a shortage of specific antibodies and/or RNA quantification tools for anti-pathogen testing. However, the data generated is not specific and has to be verified by pathogen specific methods, since contamination with other viruses or bacteria could establish a considerable bias in this setting. Moreover, the use of retrained neural networks in quantifying immunofluorescence images is comparable to other quantification methods. Notably, we also performed our analyses on a single central processing unit (CPU) and with shorter training time on a graphics processing unit (GPU). Accordingly, this approach can be used without major hardware requirements. The biological variance between experiments and other factors such as exposure time, brightfield intensity, and cell density could impact the accuracy of the neural network. Hence, we suggest to collect training images in every specific laboratory setting and from different experiments over a period of time and a variety of tissue cultures to correct for the variability observed between experiments.

In conclusion, we show the use of deep convolutional neural networks to quantify images during experimental settings of SARS-CoV-2 cultures.

## Data Availability

The source code is available at: https://github.com/MolecularMedicine2/PyQoVi. (Available from: 2 April 2021).

## Supplementary Materials

The following are available online at https://www.mdpi.com/article/10.3390/v13040610/s1, Figure S1: ResNet18 Architecture, Figure S2: ROC curve of the trained networks, Figure S3: SARS-CoV-2 infection induces CPE at day 3 post infection, Figure S4: Apoptosis assay of Vero cells stimulated with Staurosporine, Figure S5: Emetine induces toxicity and CPE in infected Vero cells, Figure S6: Drugscreening of HSP90i, Figure S7: Adjusting the Analysis of the CPE Score for Python.
