# Supplementary material for: Deep Transfer Learning Approach for Automatic Recognition of Drug Toxicity and Inhibition of SARS-CoV-2
Source: Viruses. 2021 Apr 2;13(4):610. doi: 10.3390/v13040610 (PMC8066066; doi:10.3390/v13040610)
Supplement: Supplementary file 1 [file viruses-13-00610-s001.pdf]

Figure S1

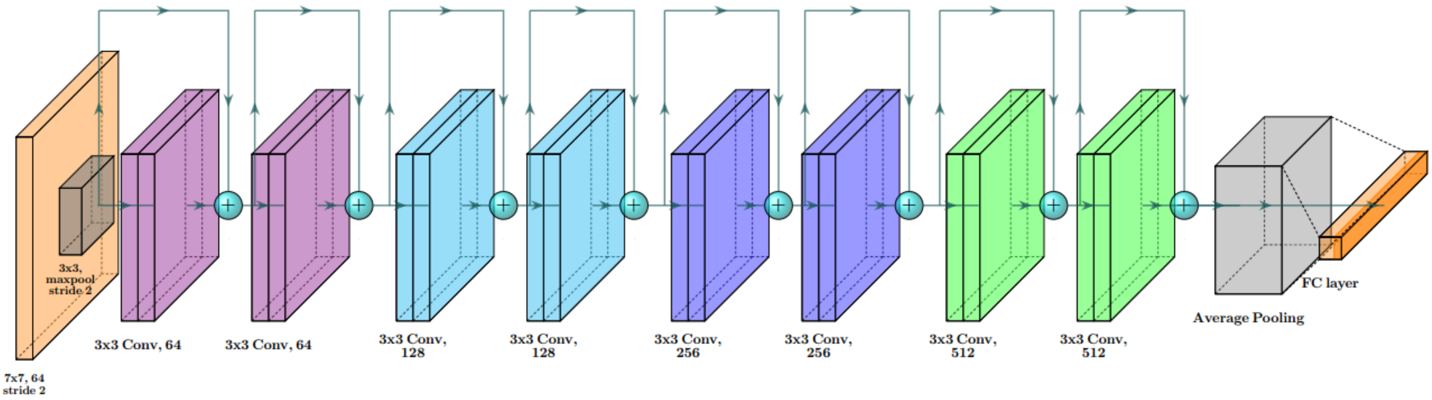

Figure S1. ResNet18 Architecture.

The Figure shows the Deep Learning architecture we used for the 'CPEnet', 'IFnet' and 'CPETOXnet' for Transfer Learning. The input shape was a 224x224x3 pixel tile.

Figure S2

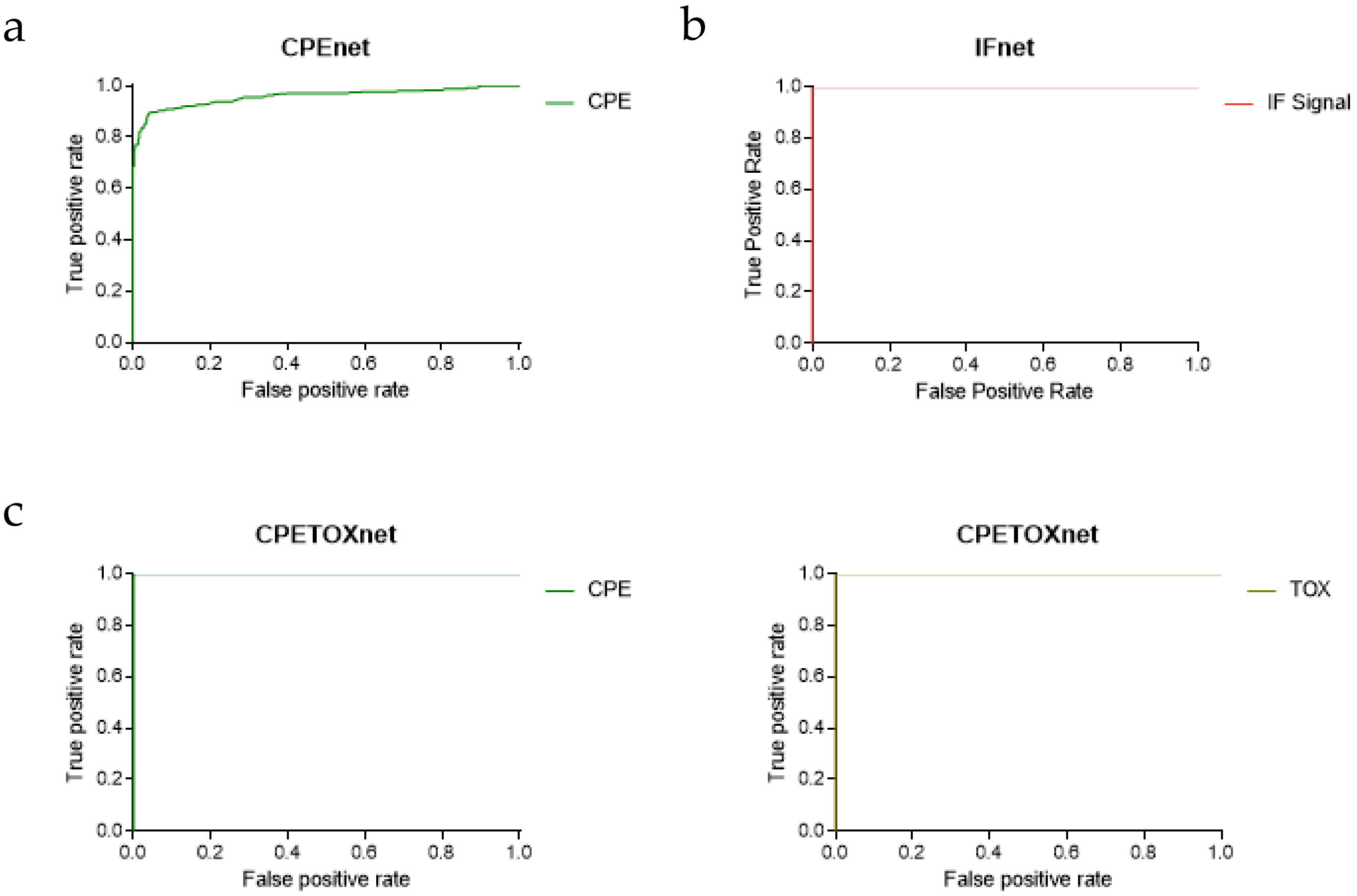

Figure S2. ROC curve of the trained netwerks.

The ROC curve of the trained (a) ‘CPEnet’ for CPE, (b) ‘IFnet’ for CPE and (c) ‘CPETOXnet’ for CPE and Tox is displayed, calculated by Matlab.

Figure S3

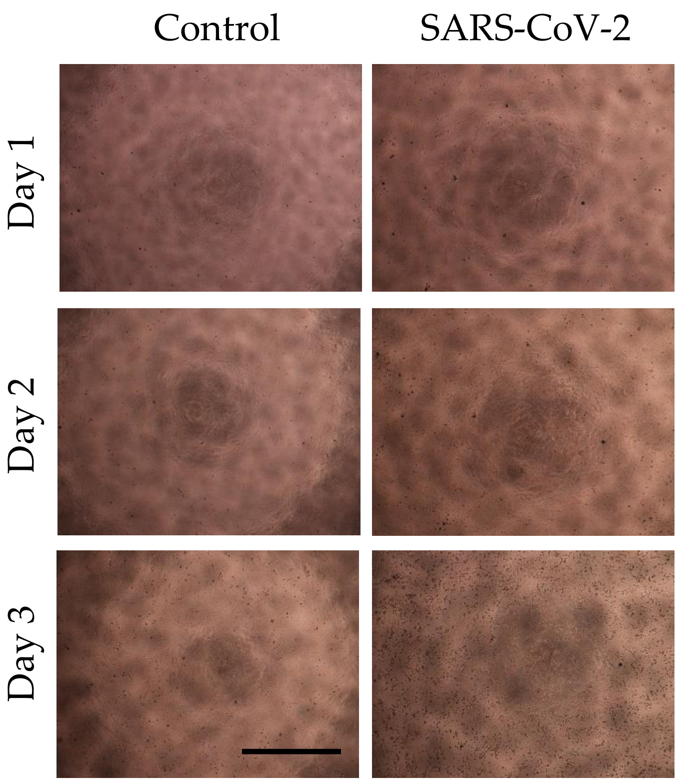

Figure S3. SARS-CoV-2 infection induces CPE at day 3 post infection.

Vero cells were infected with SARS-CoV-2 at an MOI of 0.03. Images were taken 1, 2, and 3 days after infection or without infection (scale bar = 1mm, $n = 48$).

Figure S4

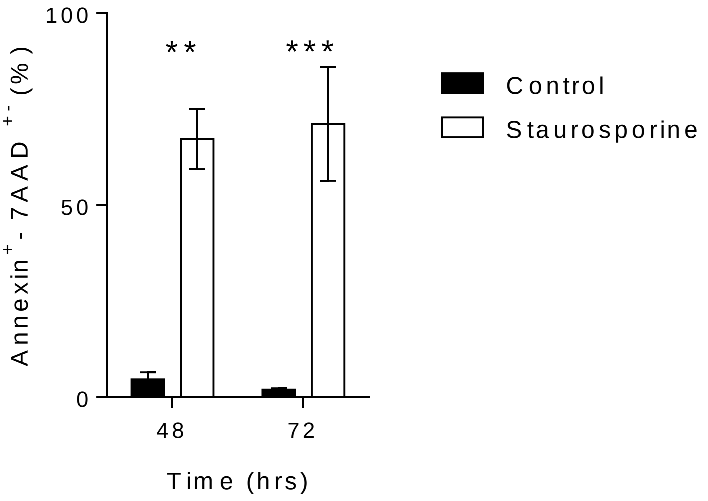

Figure S4: Apoptosis assay of Vero cells stimulated with Staurosporine.

Vero cells were treated with 5μM Staurosporine or DMSO in contol group. Graph represents AnnexinV+ 7AAD- combined with AnnexinV 7AAD double positive cells measured 48 and 72h after treatment (*n* = 3, **P<0.01, ***P<0.001).

Figure S5

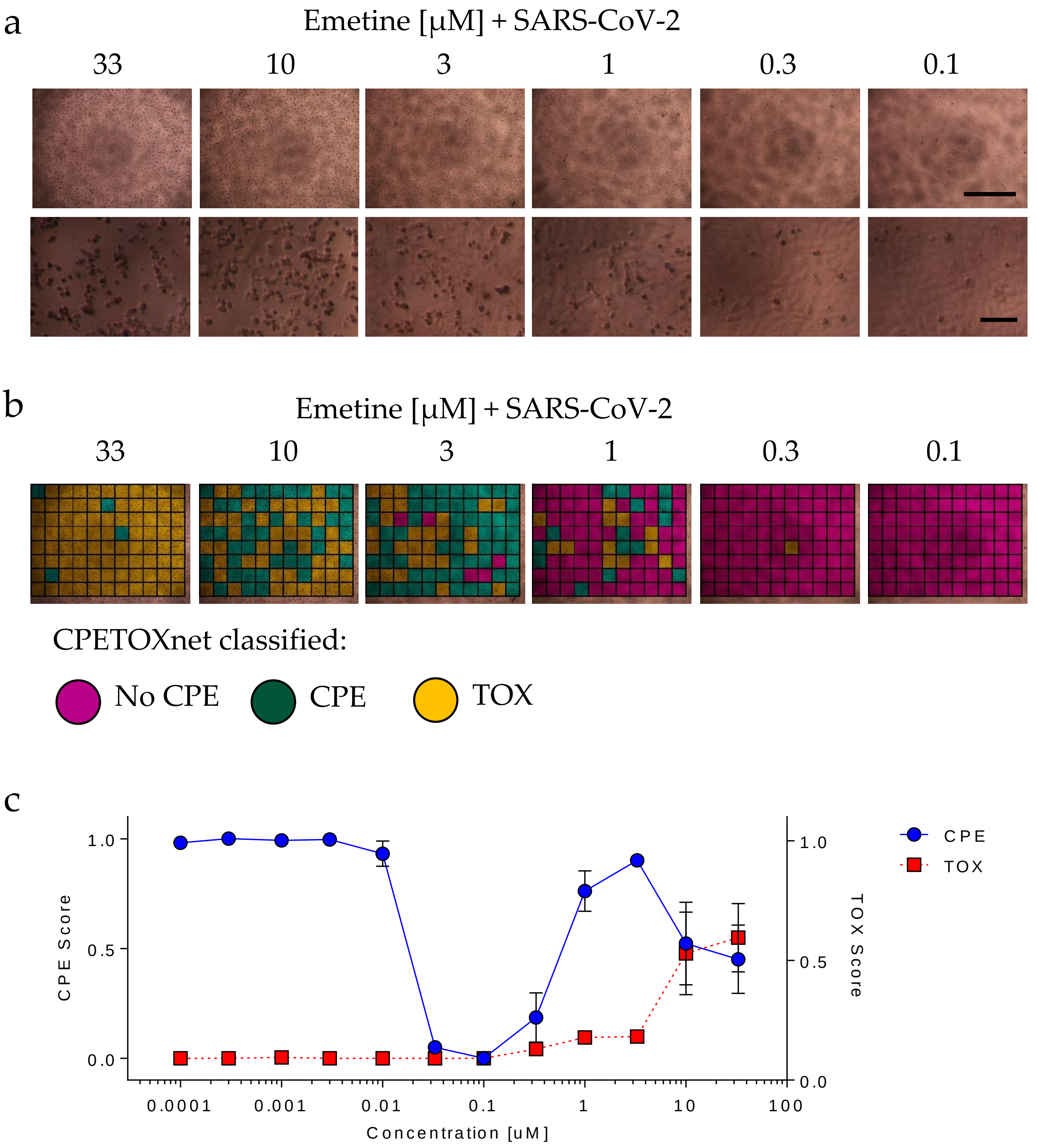

Figure S5: Emetine induces toxicity and CPE in infected Vero cells.

Vero cells were treated with the indicated concentrations of emetine and infected with SARS-CoV-2 at an MOI of 0.03. (a) 3 days post infection images were taken (one representative set of $n = 5$ is shown, scale bar = 1mm for upper panels and scale bar = 100μm for lower panels). (b) Images as in (a) were classified by 'CPETOXnet' as indicated. (c) Images were taken 3 days after infection. CPE Score (blue) and TOX Score (red) were determined by 'CPETOXnet' and are shown in a concentration dependent manner ($n = 3$).

Figure S6

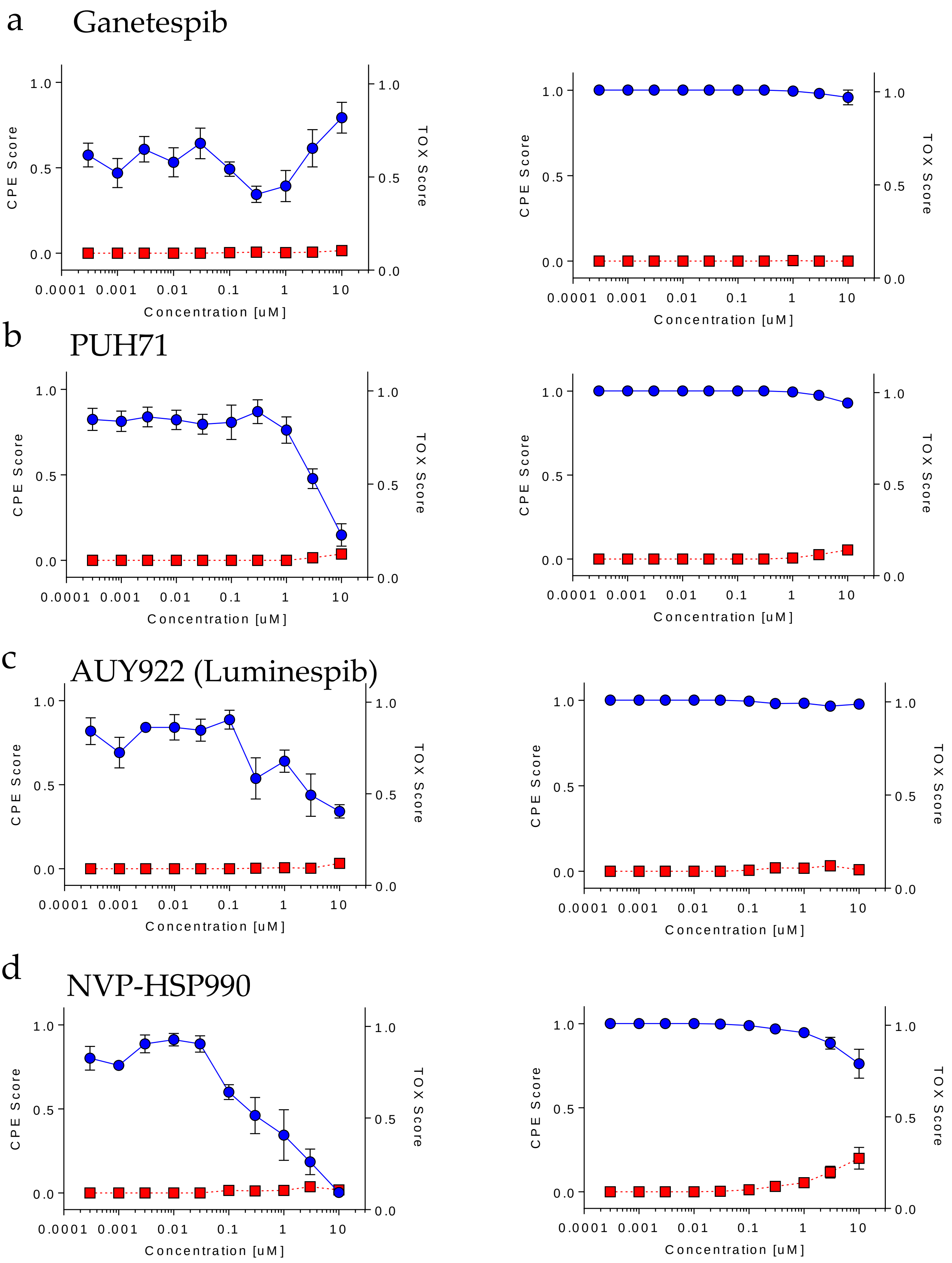

a
Ganetespib
b
PUH71
c
AUY922 (Luminespib)
d
NVP-HSP990
CPE Score
TOX Score
Concentration [uM]
0.0001
0.001
0.01
0.1
1
10
0.0
0.5
1.0

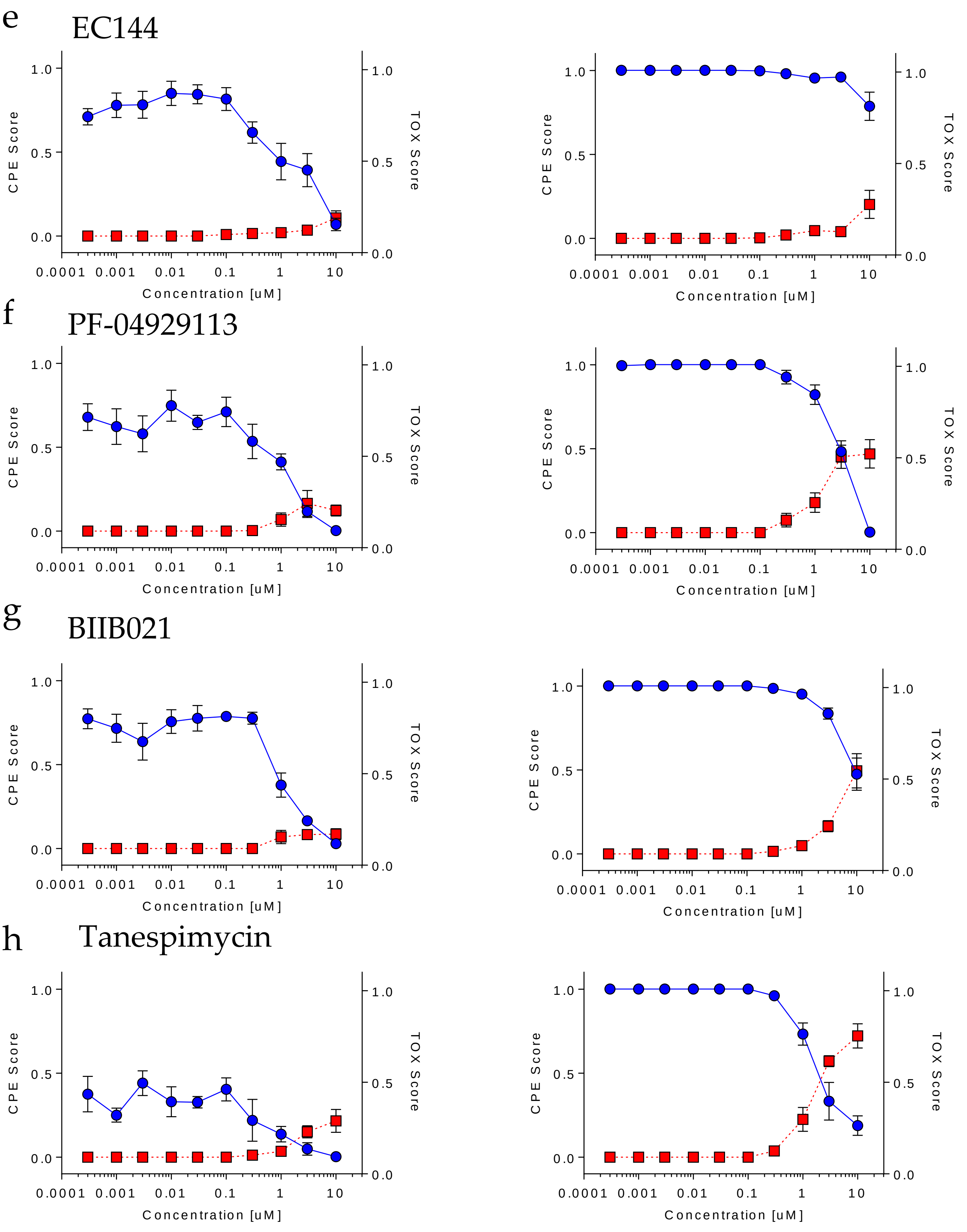

Figure S6: Drugscreening of HSP90i.

(a-g) Vero cells were treated with the indicated concentration of ganetespib (a), PUH71 (b), AUY922 (Luminespib) (c), NVP-HSP990 (d), EC 144 (e), PF-04929113 (f), BIIB021 (g) and tanespimycin (h). Images were taken 2 days (left panels), or 3 days (right panels) after infection with SARS-CoV-2 at an MOI of 0.03. CPE Scores (blue) and TOX Scores (red) were determined by 'CPETOXnet' and are shown in a concentration dependent manner ($n = 4$).

Figure S7

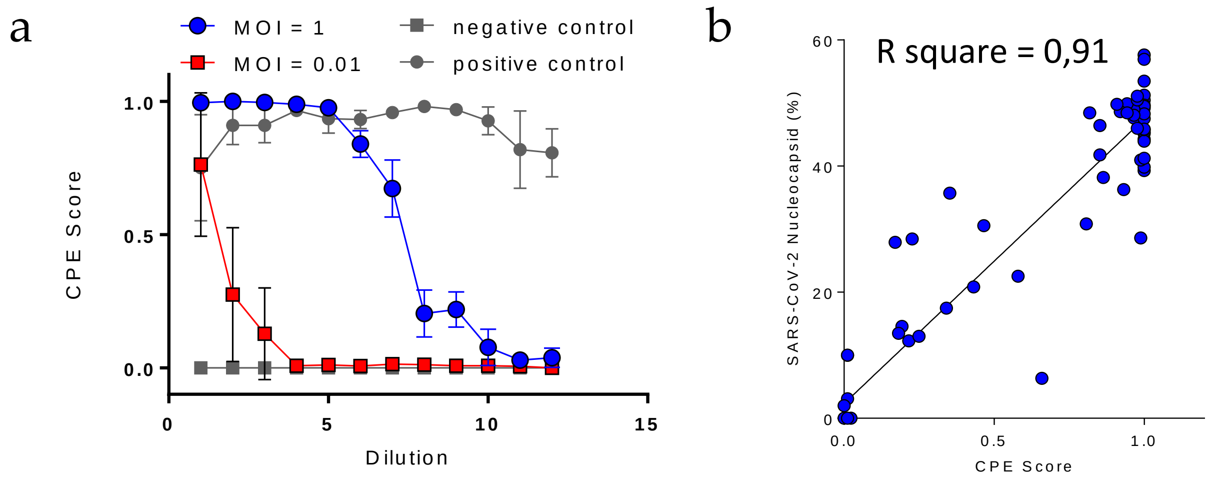

Figure S7: Adjusting the Analysis of the CPE Score for Python.

Comparison of the CPE Score analysed with Python. (a) Reanalysis of Figure 2d with the Python Deep Transfer Learning source code. Vero cells were infected with either MOI of 1 (blue line) or 0.001 (red line) of SARS-CoV-2 followed by serial 3-fold dilutions and the CPE Score was determined from bright field images. Grey closed circles indicate positive control (MOI=0.03), closed grey squares indicate negative control (*n* = 5 per well (control); *n* = 12 per well (dilution)). (b) Means of the quantification of immunofluorescence from each of 4 repeated experiments as in Figure 2e is shown as a dependence of means of 'CPEnet' analysed with Python (*n* = 76).
